# (2*E*)-2-(2-Phenyl­hydrazin-1-yl­idene)propanoic acid

**DOI:** 10.1107/S1600536811013717

**Published:** 2011-04-16

**Authors:** Md. Abu Affan, M. A. Salam, Eleazar Veronica Siew, Seik Weng Ng, Edward R. T. Tiekink

**Affiliations:** a Faculty of Resource Science and Technology, Universiti Malaysia Sarawak, 94300 Kota Samarahan, Sarawak, Malaysia; b Department of Chemistry, University of Malaya, 50603 Kuala Lumpur, Malaysia

## Abstract

The 13 non-H atoms comprising the title compound, C_9_H_10_N_2_O_2_, are close to planar (r.m.s. deviation = 0.140 Å), with maximum deviations of 0.292 (1) and 0.210 (1) Å to either side of the least-squares plane exhibited by the hy­droxy and carbonyl O atoms, respectively. The observed conformation is stabilized by an intra­molecular O—H⋯N hydrogen bond. The conformation about the N=C double bond [1.2909 (16) Å] is *E*. The hy­droxy OH group also forms an inter­molecular hydrogen bond to a carbonyl O atom, and the amine H atom similarly forms an N—H⋯O hydrogen bond to a second carbonyl O atom. The result is the formation of a double layer with a flat topology. Layers stack along the *a*-axis direction connected by C—H⋯π inter­actions.

## Related literature

For background and recent studies on the biological activity of tin/organotin compounds, see: Gielen & Tiekink (2005 ▶); Affan *et al.* (2009 ▶).
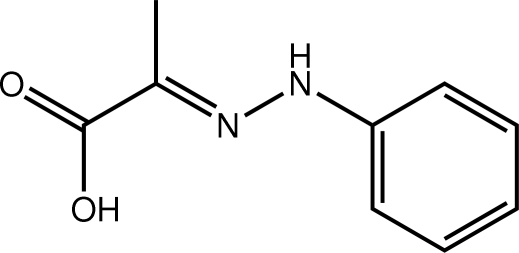


## Experimental

### 

#### Crystal data

C_9_H_10_N_2_O_2_
*M* *_r_* = 178.19
Monoclinic, 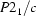

*a* = 7.3239 (3) Å
*b* = 12.0837 (7) Å
*c* = 9.6836 (4) Å
β = 99.119 (4)°
*V* = 846.17 (7) Å^3^
*Z* = 4
Mo *K*α radiation
μ = 0.10 mm^−1^
*T* = 100 K
0.20 × 0.15 × 0.10 mm

#### Data collection

Agilent Supernova Dual diffractometer with an Atlas detector
Absorption correction: multi-scan (*CrysAlis PRO*; Agilent, 2010 ▶) *T*_min_ = 0.734, *T*_max_ = 1.000
7879 measured reflections
1920 independent reflections
1544 reflections with *I* > 2σ(*I*)
*R*_int_ = 0.042

#### Refinement

*R*[*F* ^2^ > 2σ(*F* ^2^)] = 0.043
*wR*(*F* ^2^) = 0.114
*S* = 1.03
1920 reflections
127 parameters
H atoms treated by a mixture of independent and constrained refinement
Δρ_max_ = 0.21 e Å^−3^
Δρ_min_ = −0.22 e Å^−3^

### 

Data collection: *CrysAlis PRO* (Agilent, 2010 ▶); cell refinement: *CrysAlis PRO*; data reduction: *CrysAlis PRO*; program(s) used to solve structure: *SHELXS97* (Sheldrick, 2008 ▶); program(s) used to refine structure: *SHELXL97* (Sheldrick, 2008 ▶); molecular graphics: *ORTEP-3* (Farrugia, 1997 ▶) and *DIAMOND* (Brandenburg, 2006 ▶); software used to prepare material for publication: *publCIF* (Westrip, 2010 ▶).

## Supplementary Material

Crystal structure: contains datablocks global, I. DOI: 10.1107/S1600536811013717/hg5024sup1.cif

Structure factors: contains datablocks I. DOI: 10.1107/S1600536811013717/hg5024Isup2.hkl

Additional supplementary materials:  crystallographic information; 3D view; checkCIF report

## Figures and Tables

**Table 1:** Hydrogen-bond geometry (Å, °) *Cg*1 is the centroid of the C4–C9 ring.

*D*—H⋯*A*	*D*—H	H⋯*A*	*D*⋯*A*	*D*—H⋯*A*
O1—H1⋯N1	0.86 (2)	2.12 (2)	2.6169 (16)	115.9 (16)
O1—H1⋯O2^i^	0.86 (2)	2.18 (2)	2.9039 (14)	141.5 (19)
N2—H2⋯O2^ii^	0.916 (18)	2.199 (19)	3.0579 (15)	155.9 (15)
C3—H3c⋯*Cg*1^iii^	0.98	2.92	3.5830 (16)	126

Symmetry codes: (i) 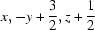; (ii) 
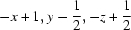
; (iii) 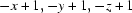.

**Fig. 1.**
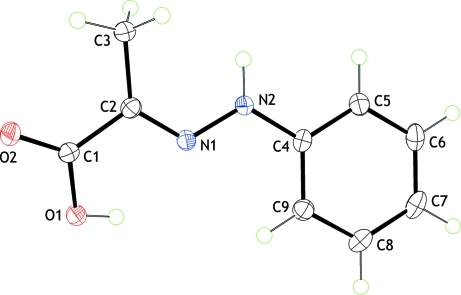
The molecular structure of (I) showing the atom-labelling scheme and displacement ellipsoids at the 50% probability level.

**Fig. 2.**
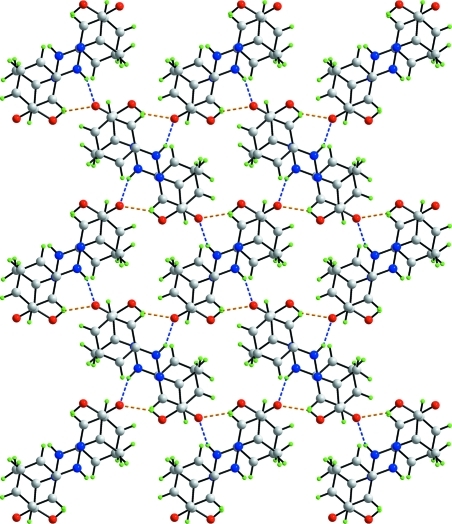
A view in projection down the a axis of the supramolecular double layer in (I). The O—H···O and N—H···O hydrogen bonds are shown as orange and blue dashed lines, respectively.

**Fig. 3.**
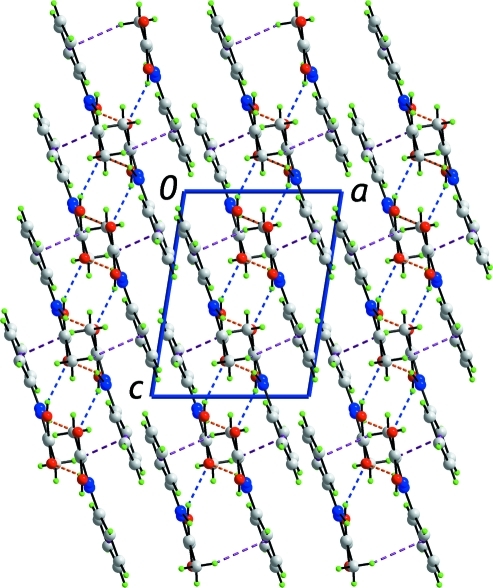
A view in projection down the b axis of the crystal packing in (I) showing the connection between layers via C—H···π interactions. The O—H···O and N—H···S hydrogen bonds are shown as orange and blue dashed lines, respectively, and the C—H···π contacts are shown as purple dashed lines.

## supplementary crystallographic information

### Comment

The title compound, (I), was prepared as a potential ligand for tin (Affan *et al.*, 2009), motivated by the wide range of biological activities displayed by organotin compounds (Gielen & Tiekink, 2005). The r.m.s. for the 13 non-hydrogen atoms comprising (I), Fig. 1, is 0.140 Å. The maximum deviations are found for the carboxylic acid-O atoms with the O1 atom being 0.292 (1) Å out of the least-squares plane and the O2 lying 0.210 (1) Å to the other side. The planarity in the molecule is readily explained in terms of an intramolecular O—H···N hydrogen bond as the hydroxy H is directed toward the centre of the molecule, Table 1. The conformation about the N1═ C2 double bond [1.2909 (16) Å] is *E*. In the crystal packing, the carbonyl-O2 atom accepts hydrogen bonds from both the hydroxy-O1—H and amine-H atoms, derived from different molecules, Table 1. The result is a supramolecular double layer as illustrated in Fig. 2. Layers stack along the *a* direction and are connected by C—H···π interactions, Fig. 3 and Table 1.

### Experimental

Pyruvic acid (0.440 g, 5 mmol) was dissolved in 10 ml absolute ethanol with constant stirring. An ethanolic solution of phenylhydrazine (0.540 g, 5 mmol) was then added to the solution drop-wise. The resulting reaction mixture was refluxed for 5 h. On cooling the solution to room temperature, a light-orange powder separated, which was filtered and washed with ethanol. The powder was recrystallized from ethanol and dried *in vacuo* over silica gel. (*M*.pt. 460–462 K. Yield 0.724 g (73.8%). Anal. Calc. for C_9_H_10_N_2_O_2_: C, 60.66; H, 5.65; N, 15.72%. Found: C, 60.61; H, 5.59; N, 15.68%. FT—IR (KBr, cm^-1^) ν_max_: 3333 (m, OH), 3285 (s, NH), 1709 (m, C=O), 1595 (w, C=N), 991 (m, N—N).

### Refinement

Carbon-bound H-atoms were placed in calculated positions (C–H = 0.95 to 0.98 Å) and were included in the refinement in the riding model approximation, with *U*_iso_(H) set to 1.2–1.5*U*_eq_(C). The O—H and N—H hydrogen atoms were freely refined; see Table 1 for bond distances.

### Crystal data

**Table:**

C_9_H_10_N_2_O_2_	*F*(000) = 376
*M**_r_* = 178.19	*D*_x_ = 1.399 Mg m^−^^3^
Monoclinic, *P*2_1_/*c*	Mo *K*α radiation, λ = 0.71073 Å
Hall symbol: -P 2ybc	Cell parameters from 2940 reflections
*a* = 7.3239 (3) Å	θ = 2.7–29.2°
*b* = 12.0837 (7) Å	µ = 0.10 mm^−^^1^
*c* = 9.6836 (4) Å	*T* = 100 K
β = 99.119 (4)°	Block, yellow
*V* = 846.17 (7) Å^3^	0.20 × 0.15 × 0.10 mm
*Z* = 4	

### Data collection

**Table:**

Agilent Supernova Dual diffractometer with an Atlas detector	1920 independent reflections
Radiation source: SuperNova (Mo) X-ray Source	1544 reflections with *I* > 2σ(*I*)
Mirror	*R*_int_ = 0.042
Detector resolution: 10.4041 pixels mm^-1^	θ_max_ = 27.5°, θ_min_ = 2.7°
ω scans	*h* = −9→9
Absorption correction: multi-scan (*CrysAlis PRO*; Agilent, 2010)	*k* = −11→15
*T*_min_ = 0.734, *T*_max_ = 1.000	*l* = −12→12
7879 measured reflections	

### Refinement

**Table:**

Refinement on *F*^2^	Primary atom site location: structure-invariant direct methods
Least-squares matrix: full	Secondary atom site location: difference Fourier map
*R*[*F*^2^ > 2σ(*F*^2^)] = 0.043	Hydrogen site location: inferred from neighbouring sites
*wR*(*F*^2^) = 0.114	H atoms treated by a mixture of independent and constrained refinement
*S* = 1.03	*w* = 1/[σ^2^(*F*_o_^2^) + (0.0516*P*)^2^ + 0.2722*P*] where *P* = (*F*_o_^2^ + 2*F*_c_^2^)/3
1920 reflections	(Δ/σ)_max_ < 0.001
127 parameters	Δρ_max_ = 0.21 e Å^−^^3^
0 restraints	Δρ_min_ = −0.22 e Å^−^^3^

### Special details

**Table:**

Geometry. All e.s.d.'s (except the e.s.d. in the dihedral angle between two l.s. planes) are estimated using the full covariance matrix. The cell e.s.d.'s are taken into account individually in the estimation of e.s.d.'s in distances, angles and torsion angles; correlations between e.s.d.'s in cell parameters are only used when they are defined by crystal symmetry. An approximate (isotropic) treatment of cell e.s.d.'s is used for estimating e.s.d.'s involving l.s. planes.
Refinement. Refinement of *F*^2^ against ALL reflections. The weighted *R*-factor *wR* and goodness of fit *S* are based on *F*^2^, conventional *R*-factors *R* are based on *F*, with *F* set to zero for negative *F*^2^. The threshold expression of *F*^2^ > σ(*F*^2^) is used only for calculating *R*-factors(gt) *etc*. and is not relevant to the choice of reflections for refinement. *R*-factors based on *F*^2^ are statistically about twice as large as those based on *F*, and *R*- factors based on ALL data will be even larger.

### Fractional atomic coordinates and isotropic or equivalent isotropic displacement parameters (Å^2^)

**Table:**

	*x*	*y*	*z*	*U*_iso_*/*U*_eq_	
O1	0.64494 (15)	0.79085 (9)	0.40334 (10)	0.0218 (3)	
O2	0.56711 (14)	0.78175 (8)	0.17390 (10)	0.0207 (3)	
N2	0.69829 (16)	0.46664 (10)	0.44283 (12)	0.0175 (3)	
N1	0.68674 (15)	0.57591 (10)	0.41916 (11)	0.0158 (3)	
C1	0.60689 (18)	0.73282 (12)	0.28486 (13)	0.0168 (3)	
C2	0.61419 (18)	0.61094 (12)	0.29678 (13)	0.0164 (3)	
C3	0.5429 (2)	0.54090 (12)	0.17314 (14)	0.0196 (3)	
H3A	0.6342	0.4838	0.1615	0.029*	
H3B	0.5208	0.5872	0.0891	0.029*	
H3C	0.4269	0.5057	0.1876	0.029*	
C4	0.78824 (18)	0.42878 (12)	0.57266 (14)	0.0163 (3)	
C5	0.7901 (2)	0.31539 (12)	0.59907 (15)	0.0205 (3)	
H5	0.7321	0.2656	0.5297	0.025*	
C6	0.87695 (19)	0.27559 (13)	0.72715 (16)	0.0242 (4)	
H6	0.8773	0.1984	0.7455	0.029*	
C7	0.9634 (2)	0.34750 (14)	0.82876 (15)	0.0248 (4)	
H7	1.0229	0.3200	0.9163	0.030*	
C8	0.96165 (19)	0.45980 (13)	0.80084 (15)	0.0228 (3)	
H8	1.0207	0.5094	0.8700	0.027*	
C9	0.87518 (19)	0.50122 (13)	0.67369 (15)	0.0193 (3)	
H9	0.8753	0.5785	0.6557	0.023*	
H1	0.656 (3)	0.7447 (18)	0.472 (2)	0.044 (6)*	
H2	0.628 (2)	0.4184 (16)	0.3834 (19)	0.032 (5)*	

### Atomic displacement parameters (Å^2^)

**Table:**

	*U*^11^	*U*^22^	*U*^33^	*U*^12^	*U*^13^	*U*^23^
O1	0.0340 (6)	0.0160 (6)	0.0142 (5)	0.0001 (4)	−0.0003 (4)	−0.0001 (4)
O2	0.0272 (5)	0.0187 (6)	0.0157 (5)	0.0014 (4)	0.0018 (4)	0.0027 (4)
N2	0.0218 (6)	0.0138 (6)	0.0157 (6)	0.0001 (5)	−0.0007 (5)	0.0008 (5)
N1	0.0166 (6)	0.0149 (6)	0.0158 (6)	0.0009 (4)	0.0026 (4)	0.0007 (4)
C1	0.0175 (7)	0.0172 (8)	0.0153 (6)	−0.0006 (5)	0.0014 (5)	−0.0006 (5)
C2	0.0159 (6)	0.0180 (8)	0.0153 (6)	−0.0003 (5)	0.0027 (5)	0.0006 (5)
C3	0.0240 (7)	0.0175 (8)	0.0162 (6)	−0.0013 (6)	0.0002 (6)	−0.0008 (5)
C4	0.0146 (6)	0.0195 (8)	0.0153 (6)	0.0022 (5)	0.0043 (5)	0.0032 (5)
C5	0.0209 (7)	0.0181 (8)	0.0223 (7)	0.0016 (6)	0.0024 (6)	0.0010 (6)
C6	0.0226 (7)	0.0211 (8)	0.0288 (8)	0.0042 (6)	0.0034 (6)	0.0088 (6)
C7	0.0195 (7)	0.0345 (9)	0.0194 (7)	0.0039 (6)	0.0005 (6)	0.0093 (6)
C8	0.0189 (7)	0.0302 (9)	0.0182 (7)	−0.0008 (6)	−0.0003 (6)	0.0014 (6)
C9	0.0188 (7)	0.0195 (8)	0.0196 (7)	−0.0013 (5)	0.0025 (6)	0.0010 (6)

### Geometric parameters (Å, °)

**Table:**

O1—C1	1.3358 (16)	C4—C9	1.390 (2)
O1—H1	0.86 (2)	C4—C5	1.393 (2)
O2—C1	1.2205 (16)	C5—C6	1.3871 (19)
N2—N1	1.3405 (16)	C5—H5	0.9500
N2—C4	1.4005 (17)	C6—C7	1.388 (2)
N2—H2	0.916 (18)	C6—H6	0.9500
N1—C2	1.2909 (16)	C7—C8	1.383 (2)
C1—C2	1.478 (2)	C7—H7	0.9500
C2—C3	1.4913 (18)	C8—C9	1.3858 (19)
C3—H3A	0.9800	C8—H8	0.9500
C3—H3B	0.9800	C9—H9	0.9500
C3—H3C	0.9800		
			
C1—O1—H1	107.9 (14)	C9—C4—N2	121.60 (13)
N1—N2—C4	118.90 (11)	C5—C4—N2	118.39 (12)
N1—N2—H2	120.4 (11)	C6—C5—C4	119.65 (14)
C4—N2—H2	119.5 (11)	C6—C5—H5	120.2
C2—N1—N2	119.05 (12)	C4—C5—H5	120.2
O2—C1—O1	119.34 (13)	C5—C6—C7	120.65 (14)
O2—C1—C2	123.52 (12)	C5—C6—H6	119.7
O1—C1—C2	117.13 (11)	C7—C6—H6	119.7
N1—C2—C1	113.77 (12)	C8—C7—C6	119.11 (13)
N1—C2—C3	126.28 (13)	C8—C7—H7	120.4
C1—C2—C3	119.95 (11)	C6—C7—H7	120.4
C2—C3—H3A	109.5	C7—C8—C9	121.11 (14)
C2—C3—H3B	109.5	C7—C8—H8	119.4
H3A—C3—H3B	109.5	C9—C8—H8	119.4
C2—C3—H3C	109.5	C8—C9—C4	119.46 (14)
H3A—C3—H3C	109.5	C8—C9—H9	120.3
H3B—C3—H3C	109.5	C4—C9—H9	120.3
C9—C4—C5	120.01 (13)		
			
C4—N2—N1—C2	175.73 (12)	C9—C4—C5—C6	0.8 (2)
N2—N1—C2—C1	179.08 (12)	N2—C4—C5—C6	−179.52 (13)
N2—N1—C2—C3	−1.5 (2)	C4—C5—C6—C7	−0.6 (2)
O2—C1—C2—N1	169.64 (13)	C5—C6—C7—C8	0.1 (2)
O1—C1—C2—N1	−10.90 (18)	C6—C7—C8—C9	0.1 (2)
O2—C1—C2—C3	−9.8 (2)	C7—C8—C9—C4	0.2 (2)
O1—C1—C2—C3	169.66 (12)	C5—C4—C9—C8	−0.6 (2)
N1—N2—C4—C9	−4.0 (2)	N2—C4—C9—C8	179.71 (13)
N1—N2—C4—C5	176.34 (12)		

### Hydrogen-bond geometry (Å, °)

**Table:**

Cg1 is the centroid of the C4–C9 ring.

**Table:**

*D*—H···*A*	*D*—H	H···*A*	*D*···*A*	*D*—H···*A*
O1—H1···N1	0.86 (2)	2.12 (2)	2.6169 (16)	115.9 (16)
O1—H1···O2^i^	0.86 (2)	2.18 (2)	2.9039 (14)	141.5 (19)
N2—H2···O2^ii^	0.916 (18)	2.199 (19)	3.0579 (15)	155.9 (15)
C3—H3c···Cg1^iii^	0.98	2.92	3.5830 (16)	126

Symmetry codes: (i) *x*, −*y*+3/2, *z*+1/2; (ii) −*x*+1, *y*−1/2, −*z*+1/2; (iii) −*x*+1, −*y*+1, −*z*+1.
